# La Grippe

**Published:** 1890-10

**Authors:** 


### Selections and Abstracts

### LA GRIPPE.

It seems to be a combination of catarrhal fever, plus muscular rheumatism. A cablegram in the daily papers from London, is to the effect that the prominent physician, Sir Oscar Jennings, says that LaGrippe is a "bastard pulmonary rheumatism." We have ourselves been in the toils of LaGrippe; and we have evidence in favor of its being not only a bastard, but a dastard, pulmonary, cerebral and universal rheumatism. If there was a single point in our anatomy which was not jumped upon by the aforesaid bastard, it would have been difficult to find it. We were sure that Mallock's problem—"*Is life worth living?*—was solved in the negative. Our spinal cord made frantic efforts to climb up through its immediate environment and clasp hands with the Pachionian bodies in the most lofty territory of the cerebrum. The sciatic nerve seemed anxious to bathe its fevered brow in the secretion gf the fourth ventricle. We were in doubt whether we would die or go crazy; and we did not care which. Ye gods, talk about the pangs of the damned! No single soul will ever reach hades, or the country immediately adjacent thereto, which will suffer a tithe of what we did the first night LaGrippe struck us.

We received the greatest relief from ten-grain doses of acetanilid every hour, until thirty grains were taken, then in smaller doses continued thereafter at intervals sufficient to command the pain. Accompanying this drug with Liq.-Tong.-Sal., or Tongaline (Mellier), as a stimulator of the excretory organs, and an anti-rheumatic remedy as well, we in due time progressed to convalescence. Our individual experience has been of service in the treatment of well on to one hundred cases since, wherein the same remedies, modified according to the conditions, were used. We are sure that our patients were indirectly benefited by our own attack, for the reason that we felt a deeper sympathy for them and a greater anxiety to relieve them.

We feel wedded, not to LaGrippe, but to the remedies mentioned for its relief. We believe the sheet anchor of treatment to be the following formula:

Acetanilid, one dram.

Tongaline, three ounces.

Elixir of lactopeptine, three ounces.

A dessertspoonful every two to four hours is indicated, accompanied in some cases by the carbonate of soda. Convalescence is slow; the alimentary tract is more or less disturbed. More of the cases in our observation have had catarrhal disturbances of the digestive tract than of the air passages. The nervous system is particularly "rattled" and recovery of tone on its part is very slow. Tonics later are indicated.

In many cases the early administration of quinine was found to be of great disadvantage; later, however, it produced good effects.

The medical journals aud the daily press will be flooded for months to come with LaGrippe literature.

Let us hope that some definite knowledge of the germ which is unquestionably at the bottom of the trouble will be evolved.

—*Medical Mirror.*

